# How should integrated care address the challenge of people with complex health and social care needs? Emerging lessons from international case studies

**DOI:** 10.5334/ijic.2254

**Published:** 2015-09-29

**Authors:** Nick Goodwin

**Affiliations:** International Journal of Integrated Care

Around the world, health and social care systems are struggling to cope with the increasing demands for services resulting from rapidly ageing populations with multiple physical, mental, and social care needs. The search for structural or technological solutions gives emphasis to the reorientation of care systems that need to prevent ill health, support self-care, deliver care closer to people's homes, eliminate waste and duplication, and reduce the reliance on hospitals and long-term care institutions.

The rapid rise of those with complex and long-term care needs presupposes the development of more integrated delivery systems that bring together clinical and non-clinical professionals from both the health and social care sectors. It also represents a challenge in how to adopt effective strategies of health promotion and ill health prevention that can address the socio-determinants of health and so support, at the end of the life-course, more active and healthy ageing. Today's fragmented health and social care systems continue to impede solutions that might otherwise prevent deterioration in health status and/or support better quality of life. This problem appears to be particular prevalent amongst the old, the poor, the vulnerable and people from ethnic and indigenous minority groups [1].

This month *International Journal of Integrated Care* publishes a special issue on the subject from case study work undertaken in 2013 by The King's Fund in the UK with the University of Toronto in Canada from funding provided by the Commonwealth Fund in the USA. Originally published as a research summary [2] the supplement contains details of six diverse international case studies examining care to people with complex health and social care needs across Australia, Canada, the Netherlands, New Zealand, Sweden and the USA [3–8] as well as a synthesis of the emerging findings and expert commentary [9,10].

Each of the case studies was selected on the basis that it was able to demonstrate how integrated care to people with complex needs could be achieved and a mixture of documentary evidence-gathering and stakeholder interviews were undertaken to build up an in-depth picture of each case. The case studies demonstrate that it has been possible to successfully organise and provide integrated care for people with complex problems in a variety of different ways with no ‘single’ approach emerging as a best method.

The case histories show a complex and dynamic evolutionary history, but the underlying finding is that changes at the interface with service users is what seems to matter most – for example, in supporting people to self-care or live independently; in developing resilience amongst carers and family members to cope with the needs of loved ones; through the holistic and flexible manner in which a wide range of care needs were managed through teams of dedicated care professionals; and in the role of community volunteers to support friends and neighbours. All of these aspects performed best within an integrated care delivery model that had sought, over time, to promote the alignment of governance and financial incentives facilitated through organised networks of care and/or the creation of new institutional forms.

In reflecting on these case studies, a key question emerging in my mind was whether such successful examples could be replicated or used to stimulate improvements in quality of care to people with complex needs in other settings? At one level, the solutions described at a service-delivery level seemed to be quite clear in the principles of the approach necessary. For example, all had a specific focus on improving personal relationships between professionals, service users and families to promote continuity of care and build self-management capabilities. Moreover, effective care processes based on holistic care assessment, care planning, care coordination and case management were advocated but presented nothing new to what we already know.

What struck home, however, was that each case described a ‘lived experience’ where the trials and tribulations inherent in building something as complex as integrated care had built up a commitment to care that seemed to go beyond the confines of people's job descriptions and created a responsive local context and culture within which integrated care was more likely to flourish. Successful integrated care cases with complexity, it might be surmised, cannot then be replicated through the equivalent of 3D printing. They develop and survive over time yet remain vulnerable to recurrent political, regulatory, financial and organisational shifts which mean that leadership and commitment and the intangible impact of ‘culture’ plays a significant part in their longevity.

As a final point, all the case studies were seen to have a poor ability to prove the ‘value’ of their work due to the lack of monitoring and evaluation of their interventions, particularly in terms of understanding improvements in the user experience. This inability to effectively measure and monitor outcomes in integrated care is, in part, down to their very complexity and the difficulties that exist in establishing measures and designing evaluations that can provide such evidence. The inability to demonstrate how approaches to integrated care can improve quality of care and be cost-effective is a major challenge and currently the Achilles heel of the integrated care movement. Investment in understanding research methods to effectively measure and evaluate outcomes of integrated care programmes is needed.

## References

[1] Øvretveit J (2011). Does clinical coordination improve quality and save money?.

[2] Goodwin N, Dixon A, Anderson G, Wodchis W (2014). Providing integrated care for older people with complex needs. Lessons from seven international case studies.

[3] McNab J, Gillespie JA (2015). Bridging the chronic care gap: HealthOne Mt Druitt, Australia. International Journal of Integrated Care.

[4] MacAdam M (2015). PRISMA: Program of Research to Integrate the Services for the Maintenance of Autonomy. A system-level integration model in Quebec. International Journal of Integrated Care.

[5] Glimmerveen L, Nies H (2015). Integrated community-based dementia care: the Geriant model. International Journal of Integrated Care.

[6] Carswell P (2015). Te Whiringa Ora: person-centred and integrated care in the Eastern Bay of Plenty, New Zealand. International Journal of Integrated Care.

[7] Andersson Bäck M, Calltorp J (2015). The Norrtaelje model: a unique model for integrated health and social care in Sweden. International Journal of Integrated Care.

[8] Kodner D (2015). Managing high-risk patients: the Mass General care management programme. International Journal of Integrated Care.

[9] Wodchis WP, Dixon A, Anderson GM, Goodwin N (2015). Integrating care for older people with complex needs: key insights and lessons from a seven-country cross-case analysis. International Journal of Integrated Care.

[10] Ashton T (2015). Implementing integrated models of care: the importance of the macro-level context. International Journal of Integrated Care.

